# Millimeter‐wave emissivity as a metric for the non‐contact diagnosis of human skin conditions

**DOI:** 10.1002/bem.22074

**Published:** 2017-08-24

**Authors:** Amani Yousef Owda, Neil Salmon, Stuart William Harmer, Sergiy Shylo, Nicholas John Bowring, Nacer Ddine Rezgui, Mamta Shah

**Affiliations:** ^1^ Manchester Metropolitan University Manchester United Kingdom; ^2^ University of Chichester Chichester United Kingdom; ^3^ Usikov Institute of Radiophysics and Electronics National Academy of Sciences of Ukraine Kharkiv Ukraine; ^4^ Royal Manchester Children's Hospital Manchester United Kingdom

**Keywords:** permittivity, eczema, malignant lesions, vascularization, burns

## Abstract

A half‐space electromagnetic model of human skin over the band 30–300 GHz was constructed and used to model radiometric emissivity. The model showed that the radiometric emissivity rose from 0.4 to 0.8 over this band, with emission being localized to a layer approximately one millimeter deep in the skin. Simulations of skin with differing water contents associated with psoriasis, eczema, malignancy, and thermal burn wounds indicated radiometry could be used as a non‐contact technique to detect and monitor these conditions. The skin emissivity of a sample of 30 healthy volunteers, measured using a 95 GHz radiometer, was found to range from 0.2 to 0.7, and the experimental measurement uncertainty was ±0.002. Men on average were found to have an emissivity 0.046 higher than those of women, a measurement consistent with men having thicker skin than women. The regions of outer wrist and dorsal forearm, where skin is thicker, had emissivities 0.06–0.08 higher than the inner wrist and volar forearms where skin is generally thinner. Recommendations are made to develop a more sophisticated model of the skin and to collect larger data sets to obtain a deeper understanding of the signatures of human skin in the millimeter wave band. Bioelectromagnetics. 38:559–569, 2017. © 2017 The Authors. *Bioelectromagnetics* published by Wiley Periodicals, Inc.

## INTRODUCTION

Skin is the largest organ of the human body, playing important roles in temperature and water regulation. In contact with the environment, it suffers a variety of damage: cancer arising from exposure to UV radiation; thermal burns from sources of heat; psoriasis and eczema from exposure to chemicals, and through allergies. However, in response to this damage, the skin presents signatures that can be measured using non‐contact millimeter wave sensors that could indicate the type and degree of the damage. This paper begins to explore those opportunities. Radiation in the millimeter wave band [Wiltse, 1984; Goldsmith et al., 1993] (30–300GHz) is ideally suited to measurement of the skin as it interacts strongly and only with the human body's skin layers [Gandhi and Riazi, 1986; Alekseev and Ziskin, 2007; Zhadobov et al., 2011; Smulders, 2012], thus enabling the potential for highly localized measurements. Furthermore, as the method presented in this paper does not involve artificial man‐made sources of radiation, only naturally present millimeter wave emission from the environment is required. There are no health perception issues, such as those associated with ionizing radiation of x‐rays and gamma rays [Smulders, 2012]. Millimeter wave sensors are operable in vivo and through medical dressings that are transparent to millimeter wave radiation, allowing diagnosis without their painful removal [Harmer et al., 2016].

It is well known that the level of water in human skin changes as a result of damage. Water levels rise as a result of vascularization around tumors and exudates from burns, while levels fall as a result of eczema or psoriasis [Griffiths et al., 2016]. Conveniently, the water molecule has a very high dipole moment, resulting in high dielectric permittivity in the millimeter wave band, which generates a large signature that can be measured using sensors in this band [Gandhi and Riazi, 1986; Alekseev and Ziskin, 2007; Zhadobov et al., 2011]. Furthermore, the permittivity changes with frequency over the band meaning lower frequencies can probe deeper skin layers, while higher frequencies can measure surface skin.

Human skin is made of three layers: the epidermis (outer layer), dermis (inner layer), and hypodermis (fat layer) [Alekseev and Ziskin, 2007]. The thickness of the epidermis layer is on average ∼0.1 mm, but it can be up to 0.7 mm on the palms of the hand. The thickness of the dermis layer varies over the body, but it is generally between 1.0 mm and 2.0 mm [Meema et al., 1964; Alekseev and Ziskin, 2007].

A convenient metric to describe the condition of human skin is emissivity, as this can be measured relatively easily using active (i.e., radar) and passive techniques [Grum and Becherer, 1979; Ulaby et al., 1981; Siegel and Howell, 2002]. A half‐space electromagnetic model of the human skin was therefore developed to determine the emissivity of healthy skin and skin having a variety of medical conditions. The reflection coefficients for healthy skin and skin with second degree burns over the band 26.5–40 GHz were taken from Gao and Zoughi [2017] with open‐ended coaxial probe data from Alekseev and Ziskin [2007], Egot‐Lemaire and Ziskin [2011], and Smulders [2012]. Permittivity data were used from the parametric models such as the Cole‐Cole model and Debye model [Gabriel et al., 1996a,1996b; Wallace et al., 2004]. Permittivity data of the healthy skin and skin with basal cell carcinoma (BCC) were calculated in the frequency band 100–300 GHz using a two‐pole Debye model [Pickwell et al., 2004; Wallace et al., 2004; Pickwell et al., 2005].

Over the past few decades, technology in the millimeter wave band has shown steady and consistent development with an increasing number of applications. A wide variety of devices (sources, detectors, and mixers) have become available, enabling novel system architecture of imagers, non‐imaging sensors, radiometers and radars to be developed. As an imaging sensor, these systems can deliver spatial resolutions of less than the wavelength, which in the millimeter wave band is down to about 1.0 mm. The instrument chosen to measure the emissivity of skin reported in this paper was a 95 GHz radiometer. This frequency was chosen as indications [Smulders, 2012] are that radiation at this frequency interacts mostly with the top 0.4 mm layer of the skin, so it is ideally suited to the measurements of the epidermis and dermis. The emissivity of skin in wrist and forearm areas of 30 volunteers was measured and analyzed and compared with models.

## MATERIALS AND METHODS

### Human Skin Emissivity Model

**Figure 1:**
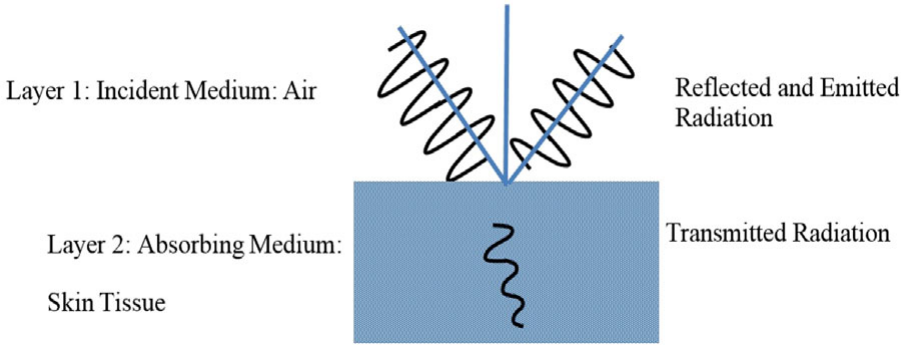
Half‐space electromagnetic model.

A simple half‐space electromagnetic model was constructed to determine the emissivity of human skin directly from measurements or simulations of either the relative complex permittivity or complex reflectivity of human tissue. In the half‐space model, one half is a semi‐infinite layer of air, and the other half is a semi‐infinite layer of skin, as shown in Figure 1.

Both layers are isotropic and homogenous and can be described by the relative complex permittivity values. Conservation of electromagnetic energy in the model gives the relationship between reflectance *R*, transmittance *T*, and emissivity *η* at the skin surface as:

(1) R+T+η=1 .

The relationship between reflectance and reflectivity (fraction of incident complex field amplitude reflected), *Γ*, can be expressed as:

(2) $$R={\left|\Gamma \right|}^{2}$$

The relationship between transmittance and transmissivity (fraction of complex field amplitude transmitted), *t*, can be expressed as:

(3) $$T={\left|t\right|}^{2}$$

In this model, it is assumed that skin is a specular reflector, which is reasonable, as the surface roughness of skin is considerably less than the wavelength of radiation. Therefore, in this half‐space model the normal and parallel polarization reflectivities of skin can be determined by the Fresnel equations [Born and Wolf, 1999] which are:

(4) $$\Gamma_{n}=\frac{\sqrt{\varepsilon_{1}}\cos(\theta_{i})-\sqrt{\varepsilon_{2}}\cos\left(\theta_{t}\right)}{\sqrt{\varepsilon_{1}}\cos(\theta_{i})+\sqrt{\varepsilon_{2}}\cos\left(\theta_{t}\right)}$$

(5) $$\Gamma_{P}=\frac{\sqrt{\varepsilon_{2}}\cos(\theta_{i})-\sqrt{\varepsilon_{1}}\cos\left(\theta_{t}\right)}{\sqrt{\varepsilon_{2}}\cos(\theta_{i})+\sqrt{\varepsilon_{1}}\cos\left(\theta_{t}\right)}$$

where *θ_i_* is the angle of incidence, *θ_t_* is the angle of transmission, $\varepsilon_{1}$ is the relative complex permittivity of medium 1, and $\varepsilon_{2}$ is the relative complex permittivity of medium 2.

The normal and parallel polarization transmissivities (likewise from the Fresnel equations) are:

(6) $$t_{n}=\frac{2\sqrt{\varepsilon_{1}}\cos\left(\theta_{i}\right)}{\sqrt{\varepsilon_{1}}\cos(\theta_{i})+\sqrt{\varepsilon_{2}}\cos\left(\theta_{t}\right)}$$

(7) $$t_{P}=\frac{2\sqrt{\varepsilon_{1}}\cos\left(\theta_{i}\right)}{\sqrt{\varepsilon_{2}}\cos(\theta_{i})+\sqrt{\varepsilon_{1}}\cos\left(\theta_{t}\right)}.$$

The penetration depth of the millimeter wave radiation in the human skin (defined as the distance over which the transmitted power reduces to a fraction of 1/*e* ^2^) is 0.782–0.23 mm over the frequency band 30–300 GHz [Gandhi and Riazi, 1986], and this is confirmed by simulations performed by the authors and presented in this paper. The short penetration depth is mainly due to the attenuating effects of water in the human body [Alekseev et al., 2008]. A consequence of this is that an accurate electromagnetic model of the skin may be realized without knowledge of deeper tissue properties. This property also enables highly localized measurements to be made of skin which cannot be obtained in the visible region of the spectrum, and so potentially constitutes the basis for a new diagnostic tool.

For an object that transmits no radiation (*T* = 0), the emissivity is equal to the fraction of the incident radiation that is absorbed, which becomes (1‐*R*) as indicated by Equation (1). In a half‐space model, therefore, the emissivity can be calculated by integrating (1‐*R*) over the air‐side hemisphere. The reflectance is a function of the polarization and the angle of incidence, and thus integration over all angles and polarization states is required to calculate the emissivity. As the illumination is isotropic and the received wave is plane, the measurements are performed in the near field zone, where the distance to the receiver (described in Experimental Method section) is 1.0 cm. The power incident over an area, dS, of skin is:

(8) $${\text{dP}}_{\text{incident}}=\text{IdS}\int_{2\pi }\cos\left(\theta \right)d\Omega$$

where *d*Ω is the solid angle that defines the direction of propagation of the radiation relative to the normal to the area dS, and *I* is the power density from the incident isotropic sources in units of watts per unit area, per steradian. Integration over the air‐side hemisphere gives:

(9) $${\text{dP}}_{incident}=I\pi \text{dS}$$

The fraction of this incident power absorbed by the skin is then

(10) $${\text{dP}}_{absorbed}=\text{IdS}\int_{2\pi }\left(1-R(\theta )\right)\cos\left(\theta \right)d\Omega =I\pi \text{dS}\left(1-\int_{0}^{\pi \setminus 2}R(\theta )\sin\left(2\theta \right)d\theta \right)$$

The emissivity *η* is the fraction of the incident radiation that is absorbed; hence, from Equations (9) and (10) this is:

(11) $$\eta =1-\int_{0}^{\pi \setminus 2}R(\theta )\sin\left(2\theta \right)d\theta .$$

In the case of unpolarized sources, the reflectance *R*(*θ*) must be replaced by the average of the normal and parallel polarization reflectances.

(12) $$R\left(\theta \right)=\frac{1}{2}\;\left[\left(Rn\left(\theta \right)+Rp\left(\theta \right)\right)\right]$$

Equation (11) provides a relationship between the emissivity and the complex permittivity of the sample. The integral in Equation (11) is not easily evaluated analytically and so a numerical approach, implemented in Matlab, was used to compute emissivity values. Equations (1) to (12) were evaluated numerically using an algorithm developed by the authors in the language of Matlab.

The uncertainties in the simulated emissivity values can be determined by error propagation of uncertainties in the relative complex permittivity values through Equations (1) to (12). Given the uncertainty in the permittivity from the single relaxation Debye model is ±0.05 [Gabriel et al., 1994; Gabriel et al., 1996a; Gabriel and Peyman, 2006; Sasaki et al., 2014], error propagation indicates the uncertainty in the emissivity of skin is ±0.006, unless otherwise stated; this is the case throughout the paper. With typical values of emissivity ranging from 0.4 to 0.75, this indicates a precision of less than ∼1.5%.

### Experimental Method to Measure Human Skin Emissivity

Human skin emissivity can be measured using a radiometer [Harmer et al., 2016]. The output of such a device is a voltage V in volts, expressed as:

(13) $$V=\alpha \left(T_{b}+T_{N}\right)$$

where *T_b_* is the radiation temperature of the source (in this case the skin) expressed in Kelvin, *α* is the receiver responsivity (in Volts per Kelvin), and *T_N_* the receiver noise temperature (also in Kelvin). However, the radiation temperature of the source can also be expressed in terms of the source emissivity *η*, skin thermodynamic (or physical) temperature *T_S_*, and background illumination radiation temperature *T*_0_ [Bardati and Solimini, 1983]:

(14) $$T_{b}=\left(1-\eta \right)T_{0}+T_{s}\eta$$

Calibration of this radiometer can be done using two black body radiator sources, one at a low temperature *T_C_* and the other at a high temperature *T_H_* [Wyatt, 1978; Ulaby et al., 1981; Pozar, 2011]. This process calibrates the receiver responsivity and receiver noise temperature, both of which are assumed to be constant; or alternatively, the system response is assumed to be linear. If the measurements are done indoors and in an anechoic environment (where there is no radiometric emission from people or lower emissions from outdoors), the low temperature calibration source can be a foam absorber at ambient temperature *T_C_*. Under these circumstances, the voltage output when measuring the low temperature calibration source becomes:

(15) $$V_{C}=\alpha \left(T_{C}+T_{N}\right)$$

and when measuring the high temperature calibration source it is:

(16) $$V_{H}=\alpha \left(T_{H}+T_{N}\right)$$

From this calibration procedure the receiver responsivity is:

(17) $$\alpha =\frac{\left(V_{H}-V_{C}\right)}{\left(T_{H}-T_{C}\right)}$$

and the emissivity from Equation (14) becomes:

(18) $$\eta =\frac{\left({V-V}_{C}\right)\left(T_{H}-T_{C}\right)}{\left({V_{H}-V}_{C}\right)\left(T_{S}-T_{C}\right)}$$

**Figure 2:**
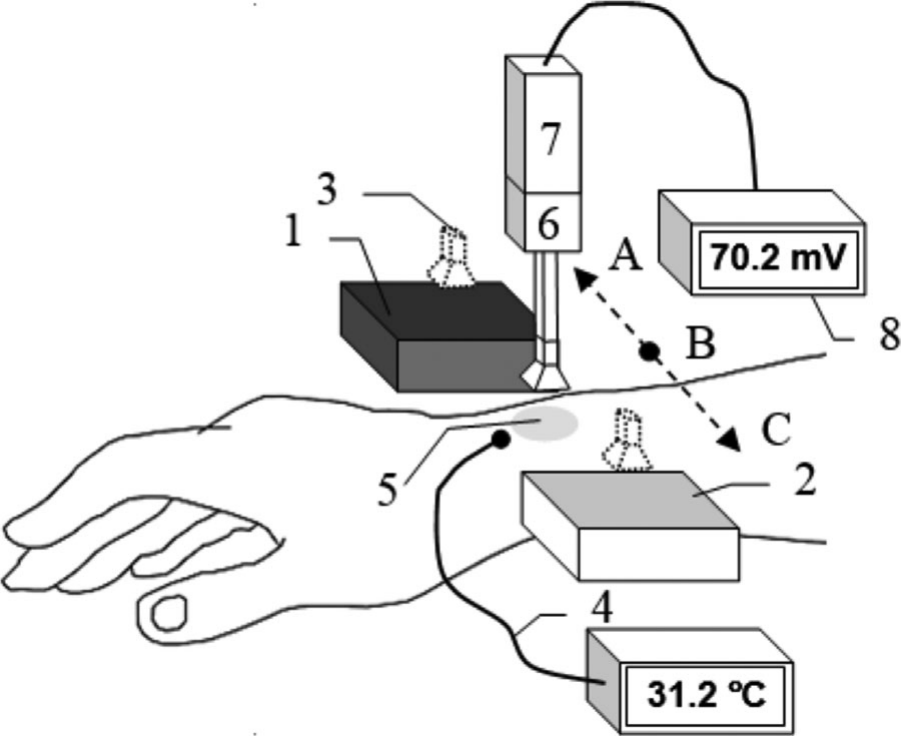
In the experimental set‐up radiometric emission at 95 GHz is collected by a moveable horn antenna (3) at positions: A to measure a hot calibration source (1), B to measure skin (5), and C to measure cold calibration source (2). A thermocouple (4) is used to measure thermodynamic temperature of skin, a digital voltmeter (8) is used to measure output voltage of calibration sources and skin. The horn antenna connected through a waveguide circulator (6) to a radiometer (7) that consists of low noise amplifier and detector.

A radiometer sensitive at 95 GHz was used for the measurements of human skin. The equipment for measurement and calibration comprised: a horn antenna connected through a circulator to a radiometer, two pieces of carbon‐loaded foam absorbers acting as hot and cold calibration sources (carbon loaded foam absorber type: Eccosorb AN‐73, Laird, Geel, Belgium), and the subject tissue to be measured, as illustrated in Figure 2.

The radiometer is essentially a low noise, high gain amplifier, followed by a detector, with sufficient sensitivity to detect the thermal (Planck) emission from ambient temperature sources over the frequency band (94–96 GHz). Radiometers have the performance metric of noise temperature measured in Kelvin; the lower the figure the more sensitive the system. The particular radiometer used for this research was purchased from Millitech (Direct Detector Radiometer, Northampton, MA). Its noise temperature was measured in the course of our research to be 453.7 K, which represents a good performance for this application.

A circulator operating over the frequency band (94–96 GHz) was purchased from ELVA‐1 Microwave Handelsbolag (CR‐10/95/2, Furulund, Sweden). The circulator was placed between the horn antenna and receiver. This device prevents radiation that has passed into the radiometer from being reflected back out of the system, which may be reflected from the subject back into the radiometer. Its function in the system was to minimize the effects of spurious signals that would otherwise arise from these retro‐reflections. In trials to identify spurious signals, by reflecting emissions from the radiometer back into the receiver, none were found.

The complete system, except for an opening for the subject to be measured, was enclosed in an anechoic region made by surrounding the majority of the radiometer and antenna with carbon‐loaded absorbing foam. This prevented radiation from external sources, be it from the outdoors or other people in the environment, getting into the system to corrupt signals.

A horn antenna has a rectangular aperture (20 × 15 mm^2^) and a nominal gain of 20 dBi, effective over the frequency band (90–100) GHz. The horn antenna was purchased from Flann Microwave (27240‐20, Cornwall, United Kingdom). During the experiment, the horn antenna was moved laterally by hand to measure emission from the subject and from the hot and cold calibration sources in relatively quick succession. It typically takes about 1.0 min to complete this measurement process, allowing for a settling time, which minimizes systematic errors associated with drift. The horn antenna during these measurements was located approximately ∼1.0 cm away from the sources, and the antenna beam pattern on the skin was approximately 20 mm across. The hot calibration source was stabilized at a temperature of 53.8 °C with a precision that was smaller than a fraction of a degree by using a Peltier plate heater/cooling device from European Thermodynamics (APH‐241‐14‐11‐E, Leicestershire, United Kingdom). The cold calibration source remained at the ambient temperature of 23.0 °C, as maintained by the building central heating system. The carbon loaded foam absorbers had emissivity values greater than 0.99 in this frequency band, thus they behaved as good approximations to a black body emitter.

Regions of the human body measured by this method were areas on the wrist and forearm. A standard thermocouple from Leaton Tech (L812, Shenzhen, China) was used to measure the skin surface temperature in these regions directly, before and after the measurement. The temperature was indicated via a digital readout with a ±0.5 °C absolute measurement uncertainty and 0.1 °C step size. An infrared thermometer from Maplin (N85FR, Manchester, United Kingdom) was used to measure the thermodynamic temperatures of the calibration sources and had an absolute measurement uncertainty of ±1.5 °C. The devices were cross‐calibrated by measuring the temperature of the same source, so the relative uncertainty of the measurement was much smaller, typically less than 0.1 °C. Typical voltage measurements were up to 100 mV with a precision of 0.1 mV. Error propagation through Equation (18) indicates the uncertainty on the measured emissivity is ±0.002.

## SIMULATION RESULTS

### Simulations of Human Skin Emissivity

Simulations of human skin emissivity were made with the half‐space model and compared to the results of an existing three‐layer model [Harmer et al., 2016], then used to predict emissivity signatures for skin with differing water contents, burned damaged skin, and skin mutated by basal cell carcinoma.

### Model Comparison: Half‐Space Versus Three‐Layer Model

**Figure 3:**
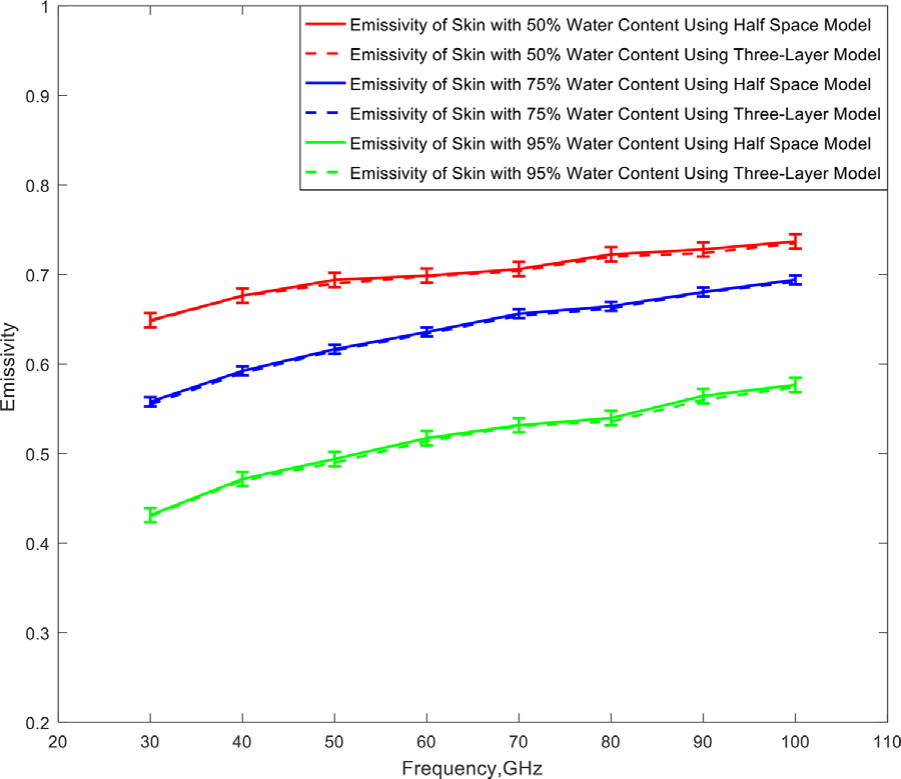
Simulations of the emissivity of human skin for the half‐space and three‐layer model show how increasing water content lowers emissivity.

The half‐space model described above (Equations (2), (3), (4), (5), (6), (7), (8), (9), (10), (11)) is a relatively simple model used to describe the emissivity of human skin. In this first simulation a comparison was made between this model and the more complex three‐layer model comprising: a semi‐infinite layer of air, 1.44 mm layer of skin, and a semi‐infinite layer of fat. The relative complex permittivity of skin was calculated from measurement of reflection coefficients of skin [Dancila et al., 2014], while the relative complex permittivity of fat tissue was calculated using Gabriel model [Gabriel et al., 1996a]. Comparison between the two models was made for skin with three different water contents, namely 50%, 75%, and 95%, and results presented over the frequency band of 30–100 GHz are in Figure 3. These results indicate that emissivity rises with frequency and falls with skin water content. The comparison shows that the results for the half‐space model are exactly the same as those for the three‐layer model.

### Skin With Differing Water Contents (Psoriasis, Normal Healthy Skin, and Malignancy)

The simulations of the emissivity of in vivo skin for the three different water contents, 50%, 75%, and 95%, shown in Figure 3, correspond to water contents that are representative of dry skin, normal healthy skin, and skin with malignant lesions, respectively [Suntzeff and Carruthers, 1946; Leunig et al., 1994; Alekseev and Ziskin, 2007; Earle, 2016]. The emissivity of skin with a 95% water content is lower by ∼0.12 than that of normal healthy skin with a water content of 75%. Properties and characteristics of skin with differing water contents are summarized in Table 1.

**Table 1:** Characteristics of Skin With Differing Water Contents

Parameters	Skin type	References
Skin with 50% water content		
Skin condition	Dry: eczema and psoriasis	Earle [2016]
Complex permittivity	6.86‐j3.33 at 100 GHz	Dancila et al. [2014]
Return loss (S11)	−9.22 dB at 100 GHz	Dancila et al. [2014]
Skin with 75% water content		
Skin condition	Healthy skin	Alekseev and Ziskin [2007]
Complex permittivity	7.34‐j5.71 at 100 GHz	Dancila et al. [2014]
Return loss (S11)	−8.59 dB at 100 GHz	Dancila et al. [2014]
Skin with 95% water content		
Skin condition	Skin with malignant lesion	Leunig et al. [1994]
Complex permittivity	7.55‐j13.72 at 100 GHz	Dancila et al. [2014]
Return loss (S11)	−5.81 dB at 100 GHz	Dancila et al. [2014]

### Skin After the Application of Aqueous Gel (30–100 GHz)

**Figure 4:**
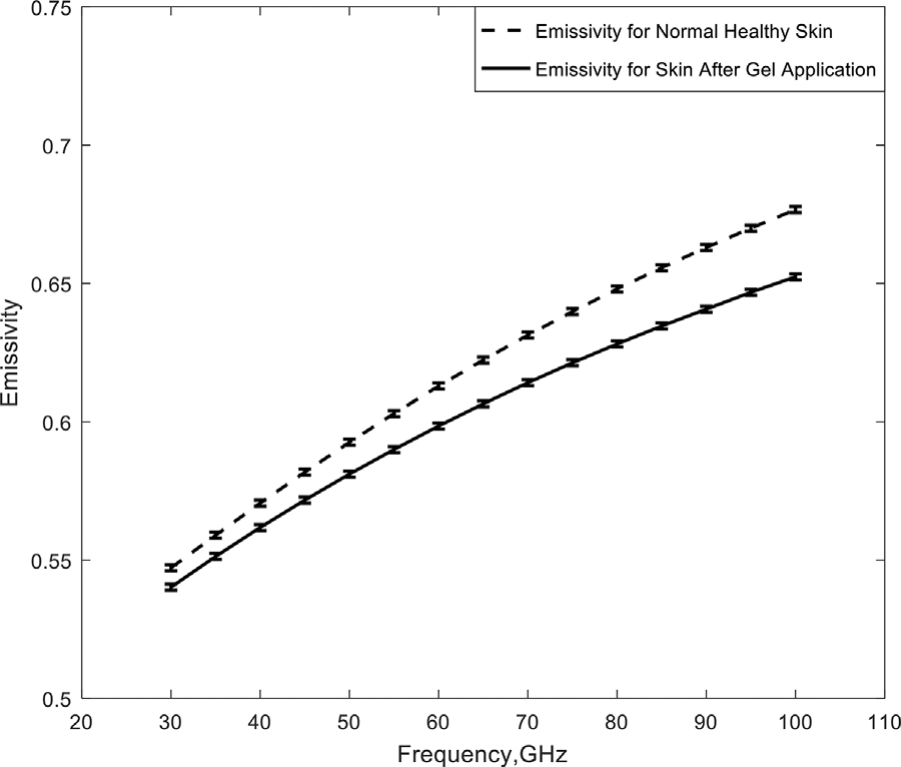
The simulated emissivity of healthy skin before and after it has been moistened by the application of an aqueous gel.

In this simulation, a comparison was made between the emissivity of normal healthy skin and skin after the application of an aqueous gel (a mixture that consists mainly of water with a thickener, such as ultrasound scan gel). This simulation used the relative complex permittivity of in vivo skin [Gabriel et al., 1996a]. The simulated emissivity rose over the frequency band from 30 GHz to 100 GHz, as shown in Figure 4, indicating emissivity is less for moistened skin, on average over the band by about 0.016. At 100 GHz this difference in emissivity rose to ∼0.025, with error propagation analysis indicating the uncertainty was ±0.0011. Aqueous gel can be used to achieve good contact between open‐ended coaxial probes and the human skin. Adding gel to the human skin increases the hydration level of the stratum corneum layer of skin and reduces the inhomogeneity of skin [Gabriel et al., 1996a; Gabriel and Peyman, 2006]. This better contact between the probe and skin reduced the systematic uncertainties in the dielectric permittivity measurements arising from the otherwise variable coupling between the probe and skin. Furthermore, the gel that consists mainly of water could be used in measurements of the dielectric properties of skin in wet state, as it can stay adhered to the skin surface longer than water. There are ethical issues associated with performing measurements on patients [Harmer et al., 2016], making the moistened skin a convenient in vivo model for damaged skin, where the damage affects the water content of the skin.

### Wet and Dry Human Skin Samples (90–100 GHz)

**Figure 5:**
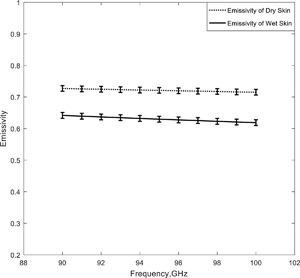
Simulations of the emissivities of samples of dry and wet human skin.

In this simulation a comparison was made between the emissivity of wet and dry skin samples taken from a human cadaver. The wet samples were taken from a 10% formaldehyde solution, rinsed in water and then measured, whereas the dry samples were dried for a 4.0 h period prior to the measurements [Alabaster, 2003]. Measurements of the relative complex permittivity were made over the frequency band 90–100 GHz [Alabaster, 2003] using free‐space method of the transmission and reflection coefficients, and these measurements were taken and used in the half‐space model to calculate the emissivity values of wet and dry skin; they are presented in Figure 5. The figure shows higher emissivity values for dry skin, with the difference being ∼0.09 ± 0.009 across the band.

### Comparison of Skin Emissivity With the Harmer Model

Emissivity calculated using the half‐space model presented here has been compared with other methods [Harmer et al., 2016]. The comparison, shown in Table 2, shows a consistent rise in emissivity with frequency, with values from this model presented here being 2.0% lower. This difference may be accounted for by the two slightly different ways in which the total reflectance is calculated. The study in this paper is a refinement on the Harmer model, integrating emission over the air‐side hemisphere, which includes reflectance at all angles from normal to glancing incidence, whereas Harmer assumes a plane wave normal to the skin surface. In the Harmer model [Harmer et al., 2016], human skin was assumed to be a lossy dielectric material and the reflection from inner layers was neglected. In this model, the relative complex permittivity measurements over the frequency band 30–37 GHz were used and converted to emissivity.

**Table 2:** Simulated Emissivity of Skin Over the 30–37 GHz Spectral Region

Frequency (GHz)	Simulated emissivity from Harmer et al. [2016]	Simulated emissivity from the half‐space model of this paper
30	0.61	0.59
31	0.62	0.61
32	0.63	0.61
33	0.64	0.62
34	0.65	0.62
35	0.66	0.63
36	0.67	0.64
37	0.68	0.65

### Burned and Unburned Porcine Skin Samples

**Figure 6:**
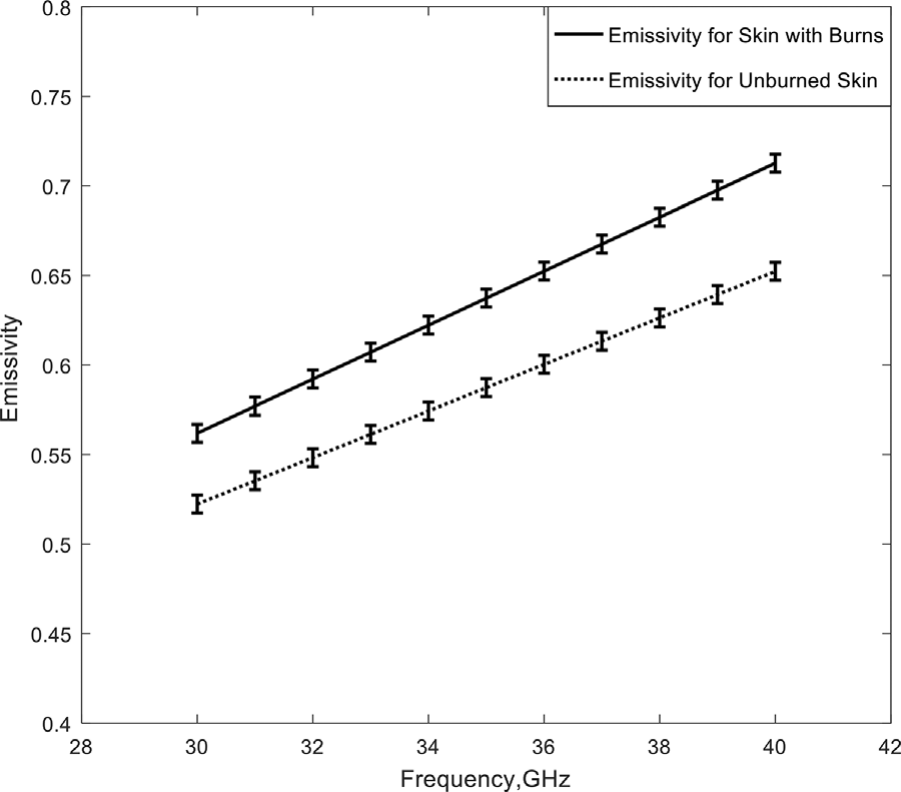
Simulations of the emissivities of unburned and second‐degree burn damaged porcine skin samples.

Simulation of the emissivity of burned and unburned skin was made using the complex reflection coefficient measurements over the frequency band 30–40 GHz [Gao and Zoughi, 2017], and the results are shown in Figure 6. In these experiments burn damage was induced in porcine skin samples after the animals had been slaughtered [Gao and Zoughi, 2017]. The simulations show a rise in emissivity with frequency and that the burned skin had an emissivity ∼0.05 higher than that of unburned skin. The absolute uncertainty is estimated to be ±0.005 from error propagation analysis.

### Skin With Basal Cell Carcinoma

**Figure 7:**
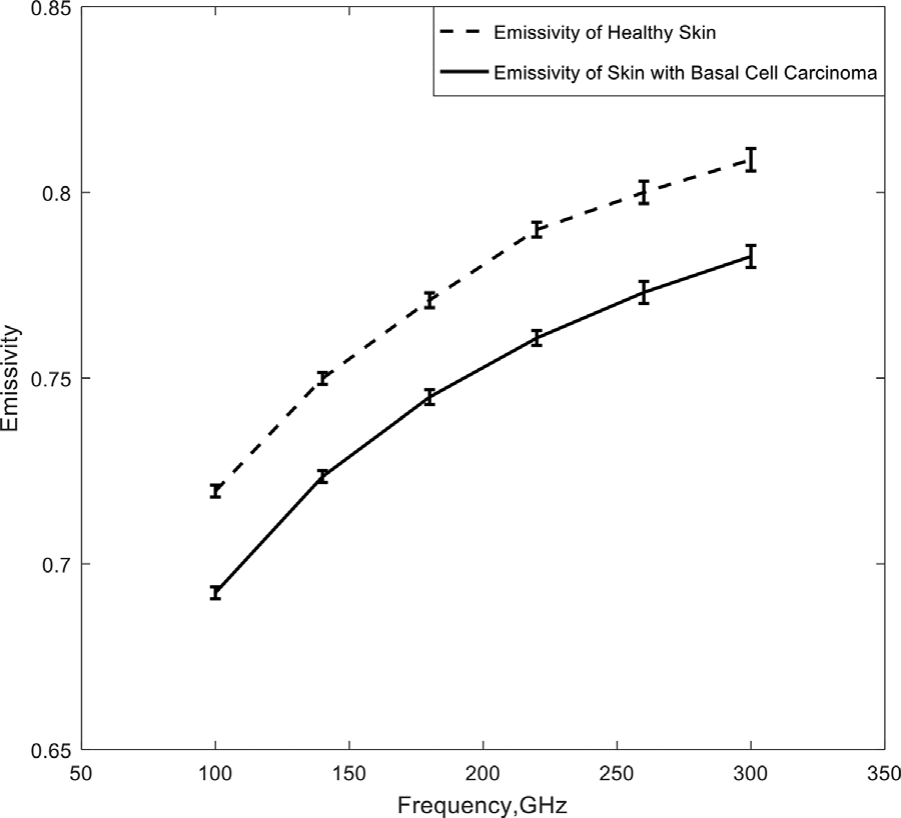
Simulations of the emissivities of healthy skin and skin with basal cell carcinoma.

The relative complex permittivity of healthy skin and skin with BCC was calculated in the frequency band 100–300 GHz using a two pole Debye model [Pickwell et al., 2004; Wallace et al., 2004; Pickwell et al., 2005]. The parameters of the model were extracted from measurements performed on five patients. From the relative complex permittivity data, the emissivity of healthy skin and skin with basal cell carcinoma was calculated using the model discussed in section two and Equation (11); results are shown in Figure 7.

The simulations indicate emissivity rises with frequency and that the emissivity of skin with basal cell carcinoma was ∼0.03 lower than that of normal healthy skin. The uncertainties in the simulated emissivity values over the frequency band were ±0.0016 (100–140 GHz), ±0.002 (150–220 GHz), and ±0.003 (230–300 GHz), estimated by error propagation analysis.

Other studies of skin with BCC using in vivo reflectivity measurements [Taeb et al., 2013] have estimated the relative permittivity to be 15.0‐j20.5 at 42 GHz, and that of healthy skin to be 11.5‐j14.3. Using these permittivity values in the above half‐space model gives emissivity values of 0.52 and 0.57 for skin with BCC and healthy skin, respectively.

## EXPERIMENTAL RESULTS

### Measurements of Human Skin Emissivity

**Figure 8:**
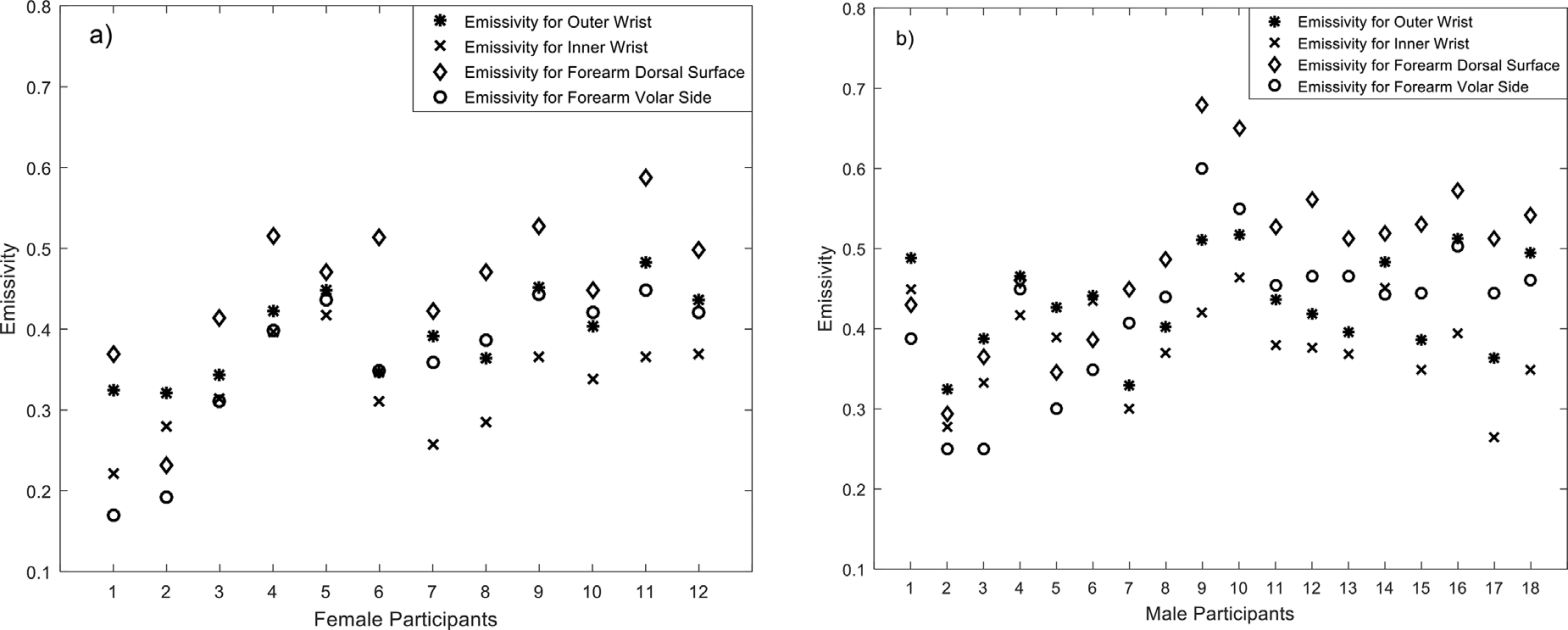
Emissivity of human skin measured at 95 GHz from 12 female (**a**) and 18 male (**b**) volunteers at four different locations on the body.

Measurements of human skin emissivity of 12 female and 18 male healthy volunteers (having a variety of ethnicities and ages) were made at four measurement locations on the body, which were: 1) inner wrist, 2) outer wrist, 3) dorsal surface of the forearm, and 4) volar side of the forearm; these are presented in Figure 8a and b). The mean emissivity over the sample of 30 volunteers was 0.413 and standard deviation in the sample was 0.091, for a measurement uncertainty of ±0.002. Upon closer inspection, trends can be seen in these data related to gender and thickness of the skin layers for different regions of the body.

Statistical analysis on the data indicates that females have a sample mean (μ) emissivity of 0.3846 with a sample standard deviation (σ) of 0.0877, generating a standard error in the mean (σ/√n, where n is the number of samples) of 0.0253. This is considerably larger than the estimated experimental uncertainty in the measurements of ±0.002. Males have a sample mean emissivity of 0.431 with a sample standard deviation of 0.0878, generating a standard error in the mean of 0.0207. The sample means of the emissivity values of the male participants are higher by 0.0464 than those of the female participants, a difference of approximately twice the standard error. This indicates that there are consistent differences in the emissivity values of the skin between females and males.

The sample mean (over all 30 volunteers) of the differences in emissivity values between the inner and outer wrist locations is 0.06 with a sample standard deviation of 0.035, generating an error in the mean of 0.0064. This difference is 9.3 times the standard error, indicating statistically significant differences between the emissivity of the inner and outer wrist locations.

The sample mean of the differences in the emissivity values between the dorsal and volar side of the forearm is 0.08 with a sample standard deviation of 0.041, generating an error in the mean of 0.0075. This difference is 10.7 times the standard error, indicating statistically significant differences between the emissivity of dorsal and volar side of the forearm.

## DISCUSSION

As simulated emissivities from the half‐space and three‐layer model of human skin (Fig. 3) were identical (within simulation uncertainty), radiation from the skin must originate from the layer no deeper than ∼1.44 mm, as that was the upper layer skin thickness in the three‐layer model. This is consistent with the statement from Gandhi and Riazi [1986] that radiation over the band 30–300 GHz is absorbed within a distance of 0.782–0.238 mm from the surface of the skin. It also means that the simulations in this paper (using the simpler half‐space model) are sufficient to describe the electromagnetic behavior of human skin over the frequency band 30–300 GHz. These simulations also show a rise in the emissivity of skin with frequency, which is a behavior that directly results from the decrease in magnitude of relative complex permittivity of water over this frequency band. As with other authors [Zhadobov et al., 2011; Smulders, 2012], we conclude that the water content of skin dominates its electromagnetic behavior in the millimeter‐wave band.

Simulations of the emissivity of human skin over the 30–100 GHz band (Fig. 4) before and after the application of an aqueous gel indicate the gel reduced emissivity at all frequencies on average over the band by ∼0.016. Simulations of samples of wet and dry human skin indicated emissivity over the band 90–100 GHz was lower for wet skin than for dry skin by ∼0.09 (Fig. 5). The lower value for wet skin was consistent with the high relative permittivity of water resulting in higher reflectance, and therefore lower emissivity.

Simulations of the emissivity of porcine skin indicate that the burned samples had an emissivity 0.05 higher than unburned samples (Fig. 6). The interpretation here is that the burning process removes water from the skin, thereby reducing the reflectance and increasing emissivity. For living organisms, however, a burn would result in exudates (mainly water) being introduced around the wound which would reduce the emissivity of the wound site. Knowledge of this and the transparency of bandages in the millimeter wave region has led [Harmer et al., 2016] to investigate of the feasibility of using millimeter waves to monitor wound healing under bandages.

Simulations of the emissivity of skin with varying water contents (50%, 75%, and 95% in Fig. 3) over the band 30–100 GHz show that emissivity rises with frequency, but falls with skin water content. This is due to the electromagnetic properties of water dominating the electromagnetic properties of skin, providing a potentially viable, non‐contact method to assist in the diagnosis of medical conditions where the skin water content is affected, such as psoriasis, eczema, and malignancy [Hagness et al., 1999; Mehta et al., 2006].

Simulations of the emissivity of human skin with basal cell carcinoma over the band 100–300 GHz indicate values were 0.03 lower than that of healthy tissue (Fig. 7). This is consistent with the interpretation that malignancy increases local vascularization, which through increased blood flow, raises the water content of tissue resulting in reduced emissivity at the site. This indicates potential opportunities of the technique for initial detection of malignancy in basal cells, which may not easily be observed in the visible band of the spectrum due to opacity of the epidermis.

Experimental measurements of the emissivity at 95 GHz from the wrists and arms of 30 volunteers indicate that there was a scatter over a range 0.2–0.7, and this was much greater than the experimental measurement uncertainty of ±0.002. Estimating the sample mean emissivity values for the 18 males and 12 females separately indicates that the difference between male and female emissivity is twice the sample standard error in the mean (Fig. 8). This finding is consistent with the skin of males being thicker than that of females [Derraik et al., 2014].

Experimental measurements of the differences in emissivity values between the inner and outer wrist, and between the dorsal and volar regions of the forearm of all 30 volunteers indicate a difference approximately 10 times the standard error of the measurement. This large difference is likely to be due to much thicker skin on the outer wrist and dorsal area of the forearm. The thinner skin on the inner wrist and volar regions [Gray, 1918] means that radiation is more readily reflected from the blood vessels [Millington and Wilkinson, 2009], and this reduces emissivity.

Simulated emissivities from the half‐space model show that radiometric emissivity rose from 0.4 to 0.8 over the frequency band 30–300 GHz, with emission being localized to a layer 1.0 mm deep in the skin. At 95 GHz the emissivity of healthy skin was simulated to be in the range 0.66–0.68, as shown in Figures 3 and 4. For comparison, the emissivity from a sample of 30 healthy volunteers, presented in Figure 8, was measured to range from 0.2 to 0.7, with the mean and standard deviation being 0.413 and 0.091, respectively. The lower values of emissivity are results of measuring particularly thin skin on the inner wrist area, whereas higher values of emissivity are results of measuring thick skin on the dorsal surface area. The spread in the emissivities also indicates the variability from person to person. The simulated emissivity values of healthy skin are in agreement with the measured values, and also in agreement with the simulations from the Harmer model [Harmer et al., 2016], as illustrated in Table 2.

The measurements indicate that differences in emissivities between thicker regions of skin (on the outer wrist and dorsal forearm) and thinner regions of skin (on the inner wrist and volar forearm) are in the range from 0.06 to 0.08. However, simulations indicate a larger variation than this for unhealthy skin, as indicated in Figures 3 and 5. It is therefore recommended that further measurements be made of the skin of participants having a range of medical conditions such as psoriasis, eczema, malignancy, and thermal burn, to better understand the effects of these conditions on emissivity.

Generally, it is recommended that further measurements be made on larger and more varied groups of individuals to study how emissivity varies with gender, age, ethnicity, and state of health. This might be done at a range of frequencies, the lower frequencies offering greater penetration into the skin and underlying tissue. It is further recommended that a more sophisticated electromagnetic model of the skin be developed to describe the complex structure of the epidermis and dermis, which might include using an electromagnetic simulation tool [Feldman et al., 2009], so that greater understanding can be made of the interpretation of future measurements.

## CONCLUSIONS

A half‐space electromagnetic model showed that emissivity of human skin rose from 0.4 to 0.8 over the 30–300 GHz band, and this behavior was consistent with the fall in the magnitude of the dielectric permittivity of water over this region. Simulations showed that the emissivity of the skin varied with water content, and this could be used as a metric to detect and monitor malignancy, eczema, psoriasis, and burn healing in skin. Simulations indicated that interaction of the millimeter waves was in the region from the skin surface to ∼1.0 mm below the surface, with greater (or less) penetration at the lower (or higher) frequencies over the 30–300 GHz band, suggesting opportunities for highly localized and selective skin layer measurements.

Radiometric measurements made on a sample of 30 volunteers at 95 GHz were consistent with the simulations presented. The measurements showed that on average the emissivity of men was higher than that of women by ∼0.0464, with standard error of the mean 0.0207 and experimental uncertainty ±0.002. This supports the knowledge that on average the skin of men is thicker than that of women [Derraik et al., 2014]. Measurements also show that the emissivity of thick layers of skin in the human body, such as the outer wrist and dorsal forearm, was higher than those of the inner wrist and volar forearm by about 0.06–0.08, this difference being approximately 10 standard errors. Again, the higher emissivity is indicative of thicker skin.

These measurements indicate the emissivity of human skin in the millimeter wave band is rich in information about skin and that these measurements can be made in tens of seconds by a non‐contact sensor with high precision. Simulations indicate this richness could be potentially exploited for the diagnosis of a range of medical conditions. Research continues in this area to understand in detail how and why emissivity varies over a much broader sample of the population of healthy volunteers and patients.
